# Supplementary material for: Is information provided within chronic subdural haematoma education resources adequate? A scoping review
Source: PLoS One. 2023 Apr 6;18(4):e0283958. doi: 10.1371/journal.pone.0283958 (PMC10079037; doi:10.1371/journal.pone.0283958)
Supplement: Attachment — Submitted filename: Review PLOS ONE.pdf [file pone.0283958.s003.pdf]

This article deals with an interesting area, that certainly needs to be known in more depth for all the aspects described in the article (first and foremost, conscious future health care choices by the patient and family and early diagnosis by non-specialist healthcare professionals).

In general, the article has many aspects that could be improved.
Next time, I recommend the inclusion of lines in the manuscript, to facilitate the review.

Abstracts

Background: it is said that CSDH is becoming prevalent, I would add a small sentence on why this is happening.

Objectives: I would remove the fact that 'they are a first step'. I would include ‘analysis and exploration’ (since you are also going to use qualitative methods and you also use an *explorative approach*).

Introduction

Third paragraph
(For CSDH this...), it is reported that neurosurgery is a third speciality, I would include more details on what this entails.
(These pose additional challenges in CSDH), I would add <u>because</u> it poses additional challenges.

Fourth paragraph
(However...) must acquire sufficient knowledge.... <u>in order to</u>... (I would add details/reasons).
(....decision-making process) I would add in '... among patients and families'.
(In CSDH....unkown) I would replace unkown with a more appropriate term such as 'poorly researched', 'little information is available about it...', as something is available and exists anyway (even if not enough).

(In this scoping...), I would change to '...we therefore explored the information in educational resources for CSDH'.
(To consider.... relatives), as the purpose of this study is not *to research themes*, but these are part of the results. I would remove this sentence and put it in the methods section.
(The review therefore....) I would recommend rephrasing the specific objectives in a clearer and more direct way. I would also include a specification of when the objective concerns patients, health professionals and when it concerns both.

Materials and Methods

First paragraph: not clearly formulated. It is unclear what is meant by *potential educational information* and which and why it is said some publication to be defined as scientific publications (are they or are they not?). It is not clear from the paragraph what is included and what is not in the review.

As specified the protocol has not been published, but can perhaps be included as supplementary information.

Search strategy
the use of a search methodology is indicated, please provide scientific references supporting its use.

Figure 1
It is not clear to the reader why the diagram is structured like this: did you start with the hospital searches that influenced subsequent searches? the diagram structured in this way could appear confusing, unless you justify why the structure is made this way (if this was done with a specific purpose). Otherwise, I would consider changing and structuring it more clearly.

Resource type acquisition and searching
Only narrative reviews were included: I would include the rationale for this choice, which to me seems to take away much of the other results potentially available in the literature.

Figure 2
It is not clear whether the words with a light grey background are readable (I printed out the figure in A4 but cannot read these words). Please evaluate the readability of this graphic.

Discussion

In general, there is not enough evidence discussed to make sense of the results obtained, links to existing literature and perspectives for future research.
What is the message this scientific article carries?
I recommend rewriting this section, so that these elements are sufficiently considered.

MMA (full word, if term not previously encountered).

Limitations and future directions

First paragraph
(Second, the search strategy....): I would give more details as to why this method was used. Also, I wondered about the criteria for this selection, e.g. were the most recent ones included?

Second paragraph
(Third, although....) I would not include this as a limitation as this is not part of the objectives of this study and therefore not a limitation (but part of a future study perhaps). If anything, it is a paragraph that could be included in the section 'perspective for future research'.

(Finally,....) I would add reasons why the fact that one of the researchers knows the topic influences the results (on what assumption is this statement based and perhaps some references that support it). As said, if this limitation is confirmed and supported, references

are needed. I would also include a sentence about how the authors tried to overcome and reduce the risk of this influence.

(This emcompass....analysis): I think this sentence is not needed here (this could be relevant to the Methods section).

(This review...process) I would state here that …in general future research perspectives in this area are....
I would not include ‘a link’ to a specific future study, which has not yet been published and about which we do not know when it will be available in scientific literature.

I would add a few sentences on the value of the results in the clinical perspective and with respect to the use of services (I recommend to reflect on this and make a connection between the results and the aforementioned areas).

Conclusion

In my opinion, it should be reformulated.
I would add what is the added value of this study and the message it carries, as well as the value of these results in a general way.

I would consider the inclusion of 'Supplementary table S1. Differences in domain components between patient orientated and healthcare professional orientated resources.' in the article itself, as this is part of the focus of the article. If preferred as supplementary, argue why and make a clear link within the manuscript on this to *invite* the reader to go and see the attached table.

All the recommended changes can make this article more readable and clear for the reader.
