# Supplementary material for: Is information provided within chronic subdural haematoma education resources adequate? A scoping review
Source: PLoS One. 2023 Apr 6;18(4):e0283958. doi: 10.1371/journal.pone.0283958 (PMC10079037; doi:10.1371/journal.pone.0283958)
Supplement: Attachment — Submitted filename: Reviewers Comments.pdf [file pone.0283958.s005.pdf]

The manuscript, following the numerous corrections made, certainly appears clearer and more readable.

I would suggest further minor adjustments as follows:

I have reported the line numbers, which refer to the corrected article (not the one with tracked changes).

Line 40: I suggest deleting 'thematic analysis,' which should be placed in the 'methods' section.

Line 91 - I suggest deleting the underlining of 'the'.

Line 122 - The second specific objective mentions the process to obtain it, not the objective itself (identify knowledge gaps, through comparison....). I would suggest rephrasing this specific objective.

Also, I do not see the overall objective of the study, which I suggest including (I can see only two specific objectives).

Line 133 - "No protocol has been published prior to this review."

I would suggest deleting this sentence, which does not add any relevant information.

Lines 126-127

Reviewer's previous comment: Only narrative reviews were included: I would include the rationale for this choice, which to me seems to take away much of the other results potentially available in the literature.

Sentence quoted in the manuscript (last version).

Narrative reviews (but not systematic reviews) were included because it was felt that they were more likely to be used by patients and were more likely to be "educational" in nature.

It is an explanation that still 'does not convince'. I would suggest explaining the reasoning made for this choice more fully and convincingly and with bibliographical references.

Discussion

- I would suggest not putting numbers and percentages in this section (as is done in the 'results' section). E.g. line 293.

  I suggest instead that terms such as 'the majority', 'one-fourth', 'more than half', 'a substantial number of...' etc. are reported in this section. The discussion should be mainly reflective in content and mainly narrative in form.

- The comment previously made by the reviewers was about reporting more reflection between the results and the literature (see previous comments from the second reviewer). I have seen that some changes have been made. I would suggest making a greater effort and adding a few additional points.

- Previous reviewer's comment: "I would add a few sentences on the value of the results in the clinical perspective and with respect to the use of services (I recommend to reflect on this and make a connection between the results and the aforementioned areas)". Although something has been added, I would ask the authors to do more thoughtful effort and expand this point in the discussion section.

I thank the authors for their important contribution of this article to the research area and suggest that these final adjustments be carried out.
