# Supplementary material for: Is information provided within chronic subdural haematoma education resources adequate? A scoping review
Source: PLoS One. 2023 Apr 6;18(4):e0283958. doi: 10.1371/journal.pone.0283958 (PMC10079037; doi:10.1371/journal.pone.0283958)
Supplement: Attachment — Submitted filename: Response to editor and reviewer comments 12.03.docx [file pone.0283958.s006.docx]

Conor S. Gillespie

Department of Clinical Neurosciences

University of Cambridge

Cambridge

CB2 0SZ, UK

12/12/2022

Dr Emily Chenette	Editor-in-Chief,	PLOS ONE

Dear Dr Chenette,

**Re: Is information provided within chronic subdural haematoma education resources adequate? A scoping review’**

Thank you considering our further revised manuscript entitled “Is information provided within chronic subdural haematoma education resources adequate? A scoping review’” for publication in PLOS One. We thank the reviewers for their constructive comments and feedback. We hope we have addressed their concerns adequately in our thorough revision of the manuscript and attach our point-by-point responses below. We strongly believe that this manuscript is an important addition to the literature and the revisions have strongly enhanced this message.

I would like to confirm again that with this submission, all study authors declare that they have reviewed the final manuscript, approved the contents of it and that the requirements for authorship have been met by all named authors. We can guarantee that the work is not being considered for publication by any other journal and that no previous work we’ve published (apart from meeting abstracts) overlap with the current work. We have not received any external funding for the completion of this work and have no relevant conflicts of interest to report. The authors received no specific funding for this work

I thank you once again for considering our manuscript for publication and look forward to hearing from you in the near future.

Yours Sincerely,

Conor S. Gillespie	Specialised Foundation Doctor (Clinical Neurosciences)	Division of Neurosurgery, Department of Clinical Neurosciences	University of Cambridge, Cambridge, UK	Email: [conorgillespie1@gmail.com](mailto:conorgillespie1@gmail.com)
**Specific Comments:**
**- Reviewer: 1**

- The manuscript, following the numerous corrections made, certainly appears clearer and more readable.

*Thank you very much for this comment- we appreciate the thorough review of the manuscript, and we hope the manuscript is more readable following these revisions.*

- Line 40: I suggest deleting 'thematic analysis,' which should be placed in the 'methods' section.

*Thank you for this comment. This has been deleted and placed in the methods section (Line 45).*

- Line 91- I suggest deleting the underlining of 'the'.

*The underlining has been deleted.*

- Line 122 - The second specific objective mentions the process to obtain it, not the objective itself (identify knowledge gaps, through comparison....). I would suggest rephrasing this specific objective.

*Thank you. This has now been rephrased to the following: “Our secondary objectives were to identify current knowledge gaps in the content of resources aimed at patients to healthcare professionals, to help in the development of future materials to facilitate shared decision making”. We hope this is sufficient and now only describes the objective itself, and not the process.*

- Also, I do not see the overall objective of the study, which I suggest including (I can see only two specific objectives).

*This has now been added (Line 116 – “The overall objective of the study was to identify the information provided in CSDH educational resources”.*

- Line 133 - "No protocol has been published prior to this review." I would suggest deleting this sentence, which does not add any relevant information.

*Thank you. This has now been deleted.*

- Lines 126-127: Reviewer's previous comment: Only narrative reviews were included: I would include the rationale for this choice, which to me seems to take away much of the other results potentially available in the literature. Sentence quoted in the manuscript (last version). Narrative reviews (but not systematic reviews) were included because it was felt that they were more likely to be used by patients and were more likely to be "educational" in nature. It is an explanation that still 'does not convince'. I would suggest explaining the reasoning made for this choice more fully and convincingly and with bibliographical references.

*Thank you. We agree that the previous statement does not explain the choice in detail. Narrative reviews were selected because they are more likely to provide educational content, as opposed to systematic reviews that adopt a focus on a specific clinical research question. The approach was based on a previously published review from the group with similar methodology, on degenerative cervical myelopathy (referenced in text Line 129). This has now been thoroughly addressed and expanded in the new methods section (Lines 127-132). We hope this now provides sufficient explanation of the choice to the reviewer.*

Discussion

- I would suggest not putting numbers and percentages in this section (as is done in the 'results' section). E.g. line 293.

*Thank you. Numbers and percentages have now been removed from the discussion section in its entirety.*

- I suggest instead that terms such as 'the majority', 'one-fourth', 'more than half', 'a substantial number of...' etc. are reported in this section. The discussion should be mainly reflective in content and mainly narrative in form.

*We agree with this and have replaced the numbers and percentages with the reviewer’s suggestion (i.e over 80% becomes over eight in ten).*

- The comment previously made by the reviewers was about reporting more reflection between the results and the literature (see previous comments from the second reviewer). I have seen that some changes have been made. I would suggest making a greater effort and adding a few additional points. Previous reviewer’s comment: “I would add a few sentences on the value of the results in the clinical perspective and with respect to the use of services (I recommend to reflect on this and make a connection between the results and the aforementioned areas)”. Although something has been added, I would ask the authors to do more thoughtful effort and expand this point in the discussion section.

*Thank you for this. We have made further changes, including a new paragraph to solely focus on this (Lines 414-421). Does the reviewer have any specific literature in mind? We have found very little with respect to shared decision making, or education in cSDH per se.*

*We hope this now represents thorough consideration and addressing of reviewer comments.*

- I thank the authors for their important contribution of this article to the research area and suggest that these final adjustments be carried out.

*Thank you for the thorough review of our manuscript. We hope that the changes recommended have been acted on appropriately, and that the latest version is now suitable for publication.*
